# Selection Based on Indirect Genetic Effects for Growth, Environmental Enrichment and Coping Style Affect the Immune Status of Pigs

**DOI:** 10.1371/journal.pone.0108700

**Published:** 2014-10-02

**Authors:** Inonge Reimert, T. Bas Rodenburg, Winanda W. Ursinus, Bas Kemp, J. Elizabeth Bolhuis

**Affiliations:** 1 Wageningen University, Department of Animal Sciences, Adaptation Physiology Group, Wageningen, The Netherlands; 2 Wageningen University, Department of Animal Sciences, Behavioural Ecology Group, Wageningen, The Netherlands; 3 Wageningen UR Livestock Research, Animal Behaviour & Welfare, Wageningen, The Netherlands; University of Florida College of Medicine, United States of America

## Abstract

Pigs living in intensive husbandry systems may experience both acute and chronic stress through standard management procedures and limitations in their physical and social environment, which may have implications for their immune status. Here, the effect of a new breeding method where pigs were selected on their heritable influence on their pen mates' growth, and environmental enrichment on the immune status of pigs was investigated. Hereto, 240 pigs with a relatively positive genetic effect on the growth of their pen mates (+SBV) and 240 pigs with a relatively negative genetic effect on the growth of their pen mates (−SBV) were housed in barren or straw-enriched pens from 4 to 23 weeks of age (n  =  80 pens in total). A blood sample was taken from the pigs before, three days after a 24 h regrouping test, and at week 22. In addition, effects of coping style, as assessed in a backtest, and gender were also investigated. Mainly, +SBV were found to have lower leukocyte, lymphocyte and haptoglobin concentrations than -SBV pigs. Enriched housed pigs had a lower neutrophil to lymphocyte (N:L) ratio and lower haptoglobin concentrations, but had higher antibody titers specific for Keyhole Limpet Hemocyanin (KLH) than barren housed pigs. No interactions were found between SBV class and housing. Furthermore, pigs with a proactive coping style had higher alternative complement activity and, in the enriched pens, higher antibody titers specific for KLH than pigs with a reactive coping style. Lastly, females tended to have lower leukocyte, but higher haptoglobin concentrations than castrated males. Overall, these results suggest that +SBV pigs and enriched housed pigs were less affected by stress than -SBV and barren housed pigs, respectively. Moreover, immune activation might be differently organized in individuals with different coping styles and to a lesser extent in individuals of opposite genders.

## Introduction

In response to stressful situations, the HPA-axis and sympathetic nervous system are activated which subsequently results in the release of glucocorticoids and catecholamines which prepare the body to fight or flight [1]. However, it is now well-known that the experience of stress also has an effect on various components of the immune system [2], [3]. For instance, one of the best known effects of acute stress is a reduction in the number of several blood leukocyte types [4]. Generally, acute stress has been suggested to enhance and chronic stress to suppress immune activation [4], although experimental results are not always that straightforward [5], [6]. The effect of stress on the immune system is suggested to be particularly mediated by glucocorticoids and catecholamines [5], [7], [8], [9].

Pigs in intensive farming systems experience acute stress during standard management procedures such as castration, tail docking, abrupt weaning, regrouping and transport [10], [11], [12], [13], [14], [15], and at the same time they have to cope with prolonged limitations in their living environment. The absence of proper substrates for oral manipulation in most intensive farming systems [16], [17] prevents pigs from performing highly motivated behaviors such as rooting and chewing [18] and may, therefore, induce chronic stress which is reflected in changes in the HPA-axis [19], [20], cognitive impairment [20], [21], [22] and in the expression of abnormal behaviors, such as tail biting and stereotypies [23], [24], [25]. Chronic stress may also be caused by ongoing social stress [26], [27]. Both short-term and prolonged stressful situations have indeed been found to influence the immune status and immune reactivity of pigs [6], [28], [29], [30], and also have major implications for pig welfare and productivity [13], [31], [32], [33].

One solution to diminish these negative side effects of stress may be provided by genetics [34], [35], [36]. For instance, social stress may be reduced by breeding pigs that perform well in group housing and do not show harmful behavior directed towards their group mates. Direct selection for pigs that perform favorable behaviors seems, however, not feasible in commercial pig breeding [34], [37], but selection on group performance is feasible and this may, indirectly, also lead to pigs with improved behavioral skills [37]. Early work of Griffing [38] and later work of Muir [39], [40] and Bijma et al. [41], [42] has shown that a phenotypical trait of an individual that lives in a group is not only influenced by its own genes, but also by the genes of its group members. This indirect genetic effect [43] on another's phenotypical trait is also referred to as associative effect [40] or social (genetic) effect [44], [45] and can relatively easy be included as a social breeding value (SBV) for production traits in commercial breeding programs [37], [42]. Hence, via indirect selection on each other's performance, animals can perform better as a group. A series of selection experiments in which laying hens were selected by taking indirect genetic effects on performance of cage mates into account, not only showed that these laying hens indeed performed better as a group, but also suggested that these hens were less sensitive to stress compared to laying hens that were selected on individual performance only [46]. Pigs can be selected for the genetic effect on each other's growth during the finishing phase [44], [45], [47] and the first results of a one generation selection experiment indicated that pigs that were selected to have a relatively positive indirect genetic effect on the growth of their pen mates (+SBV) are somewhat less fearful [48], [49] and less sensitive to stress [50] than pigs that were selected to have a relatively negative indirect genetic effect on the growth of their pen mates (−SBV). Effects of this divergent selection on indirect genetic effects for growth on immune status are so far unknown. Besides genetics, the provision of environmental enrichment is suggested to alleviate pigs from (prolonged) stress as well [17]. Environmental enrichment has, moreover, been reported to affect certain components of the immune system [51], [52].

The aim of this study was therefore to investigate both the separate and interacting effects of this new breeding method and housing on the immune status of pigs. Furthermore, the coping style of the pigs was also taken into account, because pigs with different coping styles do not only respond differently to acute and chronic stress [53], [54], [55], [56], but have also been found to differ in immune responses [57], [58]. To that aim, a contrast in prolonged stress was created by housing +SBV and −SBV pigs from 4 to 23 weeks of age in either relatively barren or straw-enriched pens. Furthermore, at 9 weeks of age all pigs were subjected to a 24 h regrouping test to induce acute stress [13]. Effects of SBV class, housing and coping style on leukocyte subsets [59], haptoglobin [60] and innate immune components [61], [62], [63], [64] were studied by taking three blood samples, i.e. before and after the regrouping test and at 22 weeks of age. We expected the +SBV pigs to be less affected by stress than the −SBV pigs which would be, subsequently, reflected in their immune status. In addition, we expected differences in immune status between pigs in barren and enriched housing as the latter are likely to suffer less from prolonged stress than barren housed pigs.

## Materials and Methods

### Ethics Statement

The experiment described in this study was approved by the Animal Care and Use Committee of Wageningen University (Protocol Number: 2010055f). Blood sampling was carried out by trained assistants and done as quickly as possible to minimize suffering.

### Animals and housing

The pigs in this study −480 in total, equally divided over five batches - were the same pigs as described in Reimert et al. [49]. In short, pigs were born at the experimental farm of Topigs Research Center IPG in Beilen, The Netherlands and reared in conventional farrowing pens. Pigs were weaned at four weeks of age and transported to the experimental farm ‘de Haar’ of Wageningen University in Wageningen, The Netherlands. Here, half of the pigs were housed in barren pens (∼1 m^2^/pig) with a partially slatted and partially concrete solid floor. Barren housed pigs received two hands of wood shavings each day from 6 weeks of age onwards. The other half of the pigs were housed in pens (∼1 m^2^/pig) enriched with 1.5 kg of straw and 12 kg of wood shavings. All pens were cleaned daily and afterwards 3 kg of fresh wood shavings and fresh straw (250 g at the start of the experiment and then gradually increased to 1.5 kg) were added to the enriched pens. In all pens, a metal chain with a ball and, from 8 weeks of age, a jute sack were attached to the wall of each pen. The jute sack was replaced when needed.

Each group of pigs consisted of three gilts and three barrows and at least two high-resisting (HR) and two low-resisting (LR) pigs (see section 2.2.1.) and groups diverged in indirect genetic effects for growth, i.e. the heritable effect on the growth of their group members. All pigs in a pen had either an estimated relatively positive indirect genetic effect (+SBV, average of 2.00 g/day) or an estimated relatively negative indirect genetic effect on the growth of their pen mates (−SBV, average of −1.62 g/day) during the finishing period (from app. 25 to 110 kg). During this period, the growth of a pig is theoretically affected by each of its pen mates which means that the total estimated indirect genetic effect on a pig's growth in this experiment was 10 g/day ((6−1) * 2.00) for the +SBV pigs and −8.1 g/day ((6−1) * −1.62) for the −SBV pigs. Pigs were obtained by mating Topigs-20 sows and Tempo boars with the most extreme positive or negative estimated indirect genetic effects for growth that were available for each batch. Direct breeding values for growth were kept as similar as possible for both SBV classes (for details about the (social) breeding value estimations see Camerlink et al., 2013). The study was approved by the Animal Care and Use Committee of Wageningen University.

### Behavioral tests

#### Backtest

Pigs were subjected to the backtest at approximately two weeks of age [48] to assess their personality or coping style [57]. In short, a piglet was put on its back for 1 min and manually restrained. During the test, the number of struggles, the latency to struggle, the number of vocalizations, and the latency to vocalize were recorded. Piglets were classified as high-resisters (HR) if they showed two struggles and produced at least 25 vocalizations, or showed at least three struggles. Low-resisting (LR) pigs were piglets that showed 0 or 1 struggle, or 2 struggles and produced less than 25 vocalizations.

#### Regrouping test

At 9 weeks of age, pigs were exposed to a regrouping test [50] which is a stressful event for pigs [13]. In short, a pair of pigs was regrouped for 24 h in a new pen with two other pairs of unfamiliar pigs. Pairs of pigs were always mixed with other pairs from the same SBV class and housing condition and the new temporary group composition was balanced for gender and coping style. After the 24 h, each pair of pigs was put in its original pen and group again.

### Blood collection and analyses

**Figure 1:**
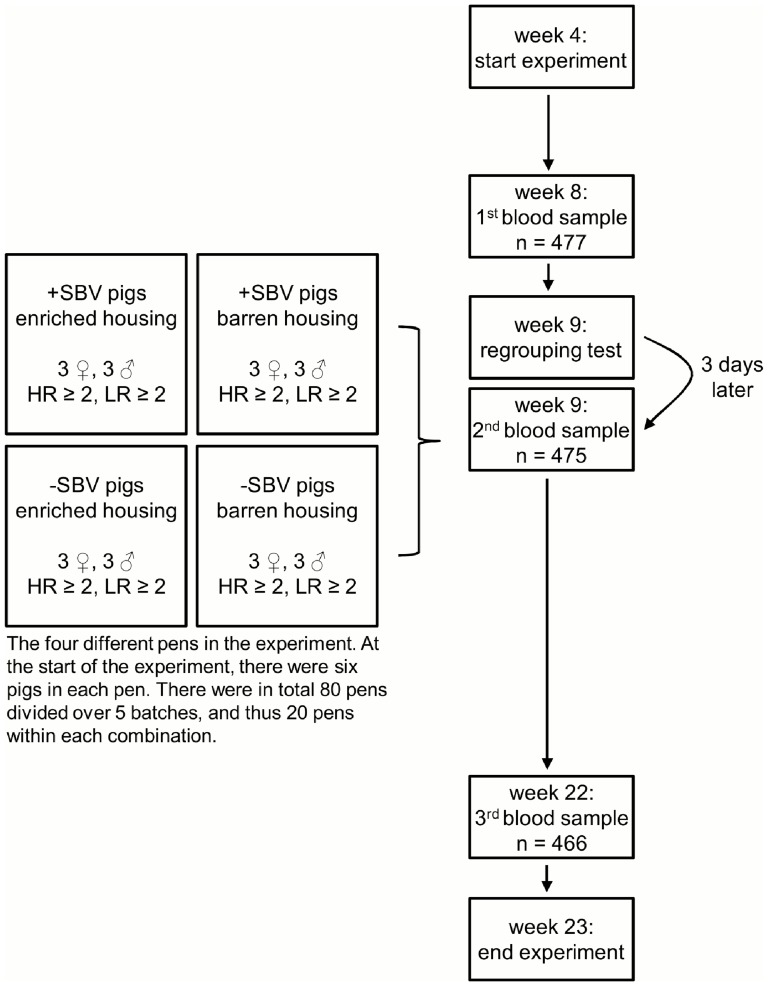
Flow diagram of the experiment. A flow diagram of the experiment showing the different treatments, the number of pigs and pens, and the number of pigs sampled at 8, 9 and 22 weeks of age.

Blood was collected from the pigs in the week before the regrouping test at 8 weeks of age, three days after the regrouping test at 9 weeks of age, and at 22 weeks of age (Figure 1). Hereto, a pig was immobilized on its back in a crib (for the first and second collection) or fixated using a nose sling (for the third collection) and blood was taken by venipuncture from the jugular vein. Housing condition and SBV class were taken into account in the order of blood collection. Blood was collected in serum separating tubes (Greiner bio-one, Kremsmünster, Austria) which were stored at room temperature (RT) and in K3 EDTA tubes (Greiner bio-one, Kremsmünster, Austria) which were stored on ice after blood sampling.

In the laboratory, the serum separating tubes were incubated for one hour at 37°C after which they were centrifuged at 5251 g for 12 min at 20°C. Obtained sera were stored at −80°C until further analysis (sections 2.3.1, 2.3.2 and 2.3.4). Blood from the EDTA tubes was used directly (section 2.3.3).

#### Complement activity via the classical (CPW) and alternative pathway (APW)

The hemolytic activity of both CPW and APW complement was measured using the hemolytic complement assay of Demey et al. [65]. In short, for CPW complement activity, 50 µl of serum was diluted serially in a gelatin-VBS-salt buffer and incubated with hemolysin sensitized sheep red blood cells for 90 min at 37°C in 96-well microtiter plates. During incubation, plates were shaken every 30 min in a Titertrek (Flow Laboratories). After 90 min, the amount of light scattered by the red blood cells upon lysis was read at 655 nm in a microplate reader (BioRad model 3550). The readings were transformed using a log-log equation [66] and the hemolytic titer was expressed as the titer that lysed 50 % of the red blood cells (CH50 U/ml). For APW complement activity, the same assay was used except that sera were diluted serially in a gelatin-VBS-EGTA buffer and incubated with rabbit red blood cells [65].

#### IgG and IgM antibody titers specific for KLH

Antibody titers of IgG and IgM specific for Keyhole Limpet Hemocyanin (KLH) [67] were determined by a two-step enzyme-linked immunosorbent assay (ELISA) similar to Bolhuis et al. [57] and Lammers et al. [67]. First, medium binding microtiter plates (Greiner Bio-one, Alphen a/d Rijn, The Netherlands) were coated overnight at 4°C with 2 mg/ml KLH in coating buffer (0.05 M Na_2_CO_3_ × 10 H_2_O, pH 9.6). After washing with tap water containing 0.05% Tween 20, serial dilutions of serum were added and incubated for one hour at RT. After washing, plates were incubated for one hour at RT with a 1:20000 diluted peroxidase (PO)-conjugated goat antibody directed to swine IgG_FC_ (GαSw-IgG_FC_/PO, Bethyl Laboratories, Montgomery, USA) to detect binding of IgG and with 1:20000 diluted peroxidase (PO)-conjugated goat antibody directed to swine IgM_FC_ (GαSw-IgM_FC_/PO, Bethyl Laboratories, Montgomery, USA) to detect binding of IgM, respectively. After washing, tetramethylbenzidine and 0.05% H_2_O_2_ were added as a substrate and incubated for 10 min at RT. The reaction was stopped with 2.5N H_2_SO_4_ and the absorbance was measured at 450 nm with a Multiskan (Flow, Irvine, UK). Each absorbance was expressed relatively to the absorbance of a standard positive control serum and antibody titers were determined as described in Schrama et al. [58].

#### Leukocytes, lymphocytes and the neutrophil to lymphocyte ratio

With 150 µl of the blood in the EDTA tubes, the concentration of leukocytes (10^9^/l) was determined with a Sysmex F-820 (Sysmex Corporation, Kobe, Japan) and with 10 µl, a smear was made on a microscope plate. After the smears had dried, they were fixed with a methanol solution and thereafter stained using a rapid staining kit (Hemacolor staining kit, Merck KGaA, Darmstadt, Germany). Surplus staining solution was washed away with PBS and then smears were dried. The percentages of lymphocytes, neutrophils, monocytes and eosinophils (the last two were not used further in this study, because of low occurrence: monocytes: 8.4 ± 0.2 %, eosinophils: 2.0 ± 0.1 % (overall mean ± SEM)) were determined by microscopic examination of the smears and counting 100 leukocytes in total using an Assistant-Counter AC-8. From these counts, the neutrophil:lymphocyte ratio (N:L ratio) was determined. Furthermore, the concentration of lymphocytes (10^9^/l) was determined by multiplying the percentage of lymphocytes with the leukocyte concentration.

#### Haptoglobin

Haptoglobin concentrations were determined in serum using a commercial kit based on the hemoglobin-binding capacities of haptoglobin (Phase Haptoglobin, Tridelta Development Limited, Maynooth, Ireland) which has been validated for pigs (GD Animal Health Service, Deventer, The Netherlands). Briefly, 100 µl of hemoglobin was added to 7.5 µl serum and solutions were gently mixed. Thereafter, 140 µl of chromogen was added and incubated for 5 min at RT. The absorbance was read immediately at 600 nm in a microplate reader. Haptoglobin concentrations (mg/ml) were calculated by using a standard linear curve with known concentrations of haptoglobin.

### Statistical analyses

SAS (SAS 9.2, SAS Institute Inc.) was used for all statistical analyses. Variables could not be obtained from all 480 pigs at each sampling period (Figure 1). This was because some pigs died during the experiment (respiratory problems, n = 3; meningitis n = 2, other causes n = 2), and some pigs were removed from the experiment because they had tail biting wounds (n = 10), were lame (n = 5), or had an umbilical cord hernia (n = 4) or rectal prolapse (n = 2). These numbers were not different for the SBV classes, housing conditions or coping styles, except that in barren housing more pigs were removed from the experiment due to a tail wound (barren vs. enriched: n = 9 vs. n = 1). In addition, missing values were present for another 20 pigs due to technical problems during blood sampling or during the laboratory assays. Moreover, for 22 other pigs the blood in the EDTA tubes had clotted after sampling which therefore became unreliable to use for further testing. Also these pigs were equally distributed across the different treatments (P ≥ 0.20). Prior to analysis, the variables CPW complement, haptoglobin, and N:L ratio were log transformed and the number of lymphocytes square root transformed to obtain normally distributed residuals. The effects of week, SBV class, housing, backtest classification and gender on the variables were assessed with a repeated linear mixed model with the fixed factors week, SBV class, housing, backtest classification, their interactions, gender, its interaction with week, and batch. Values in time of individual pens and pigs were taken as repeated measurements, i.e. SBV class, housing and batch effects were tested against the random effect of pen, and backtest classification and gender effects were tested against the random effect of pig. The order of collection within a sampling day was included as covariate.

To investigate the effect of SBV class, housing, backtest classification and gender on the variables after acute stress, the delta between week 9 and week 8 was calculated and subsequently analyzed with a linear mixed model with SBV class, housing, backtest classification, their two-way interactions, gender, and batch as fixed effects and pen, nested within SBV class, housing, and batch, as random effect. Prior to this analysis, CPW complement activity was log transformed to obtain normality of residuals.

For brevity, only significant interactions are reported. Significant interactions were further investigated with post hoc pairwise comparisons using the differences of the least square means. Results are presented as means ± SEM.

## Results

### Classical (CPW) and alternative (APW) complement activity

**Figure 2:**
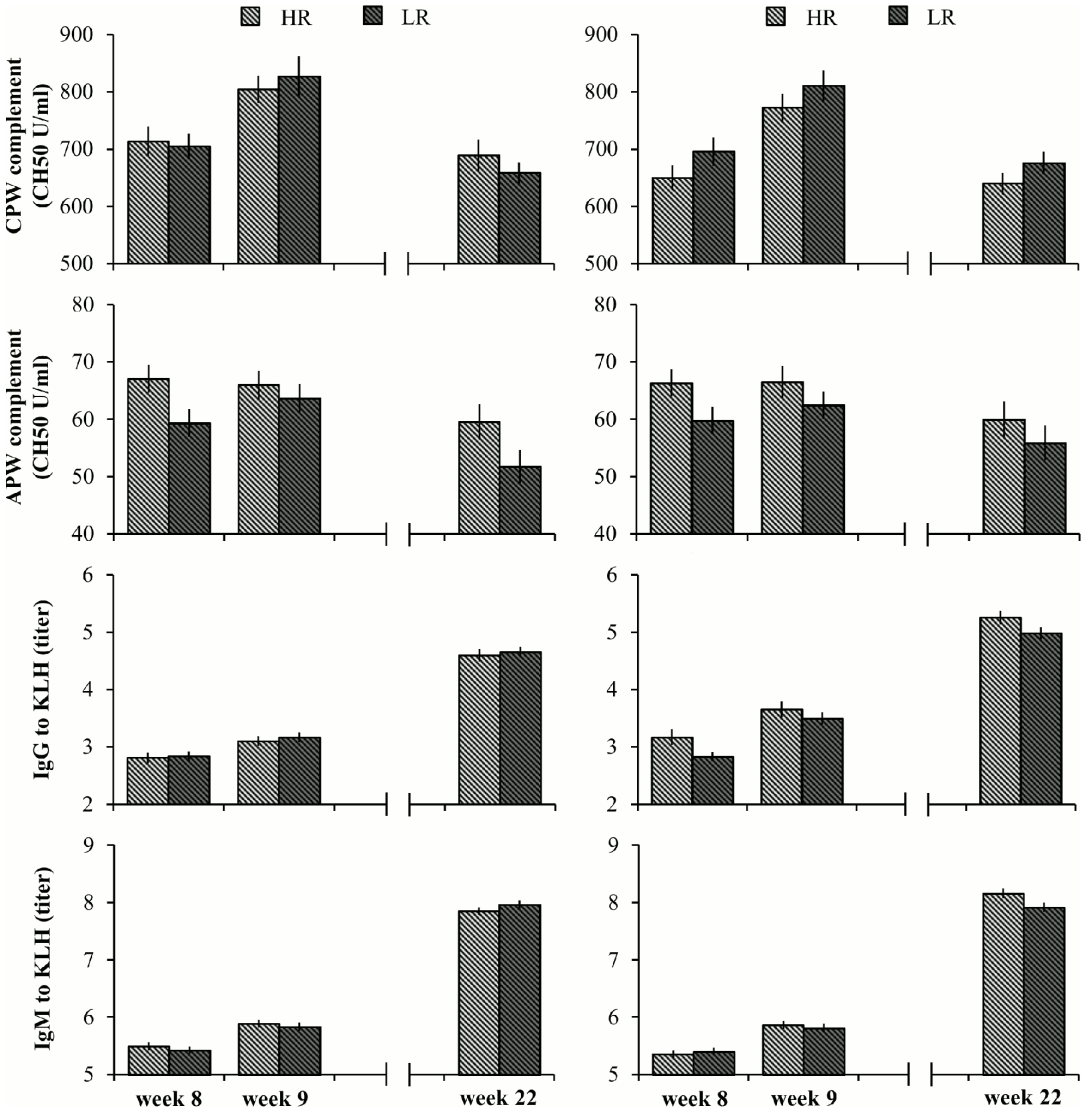
Means and SEM of complement activity and antibody titers. Compliment activity via the classical (CPW) (panels A and B) and alternative pathway (APW) (panels C and D), and IgG (panels E and F) and IgM titers (panels G and H) to Keyhole Limpet Hemocyanin (KLH) of pigs with a high-resisting (HR) and low-resisting (LR) backtest classification in barren and enriched housing measured before a 24 h regrouping test at 8 weeks of age, after the regrouping test at 9 weeks of age and at 22 weeks of age. Significance of effects of housing and backtest classification is given in the text.

CPW complement activity was not affected by SBV class, housing or backtest classification (P ≥ 0.29). There was, however, an effect of week on CPW complement activity (P <0.001) (Figure 2A and 2B), with higher activity in week 9, three days after regrouping, than in week 8 or 22. The increase in CPW complement activity from week 8 to 9 (i.e. the delta) was not affected by SBV class, housing or backtest classification (P ≥ 0.12).

APW complement activity was affected by backtest classification (P <0.01) and week (P <0.001) (Figure 2C and 2D). HR pigs had a higher APW complement activity than LR pigs (overall: 64.3 ± 1.1 vs. 58.9 ± 1.1 CH50 U/ml). In addition, APW complement activity was lower in week 22 compared to weeks 8 and 9. APW complement activity was not affected by SBV class or housing (P ≥ 0.54). Although APW complement activity did, overall, not differ between weeks 8 and 9, the delta between weeks 8 and 9 was affected by backtest classification (P <0.05). From week 8 to 9, after regrouping, APW complement activity decreased slightly for HR pigs, but increased for LR pigs (−1.0 ± 1.7 vs. 3.4 ± 1.6 CH50 U/ml). The delta in APW complement activity was not affected by SBV class or housing (P ≥ 0.93).

### IgG and IgM titers specific for KLH

KLH-IgG titers were affected by housing (P <0.001) and week (P <0.001) and by the interaction between housing and backtest classification (P <0.05) (Figure 2E and 2F). Post hoc analysis showed that enriched housed HR pigs (4.0 ± 0.1) had a higher titer than enriched housed LR pigs (3.8 ± 0.1) and that both had a higher titer than the barren housed HR and LR pigs (both: 3.5 ± 0.1). Furthermore, KLH-IgG titers increased from week 8 to 9 to 22. KLH-IgG titers were not affected by SBV class (P  =  0.43). The increase of the KLH-IgG titer from week 8 to 9 tended to be smaller for +SBV pigs than for -SBV pigs (0.37 ± 0.06 vs. 0.47 ± 0.06, P <0.1) and was larger for enriched housed pigs than for barren housed pigs (0.59 ± 0.07 vs. 0.32 ± 0.08, P <0.05), but was not affected by backtest classification (P  =  0.15).

KLH-IgM titers were affected by week (P <0.001) and the interaction between housing, backtest classification and week (P <0.001) (Figure 2G and 2H). Post hoc analysis showed that KLH-IgM titers, similar to the KLH-IgG titers, increased from week 8 to 9 to 22. Housing and backtest classification did not affect the KLH-IgM titer in weeks 8 and 9, but HR enriched housed pigs had a higher KLH-IgM titer than the other pigs in week 22. KLH-IgM titers were not affected by SBV class (P  =  0.19). The increase of the KLH-IgM titer from week 8 to 9 was not affected by SBV class, housing or backtest classification (P ≥ 0.16).

Leukocytes, lymphocytes and the ratio of neutrophils to lymphocytes (N:L ratio).

**Figure 3:**
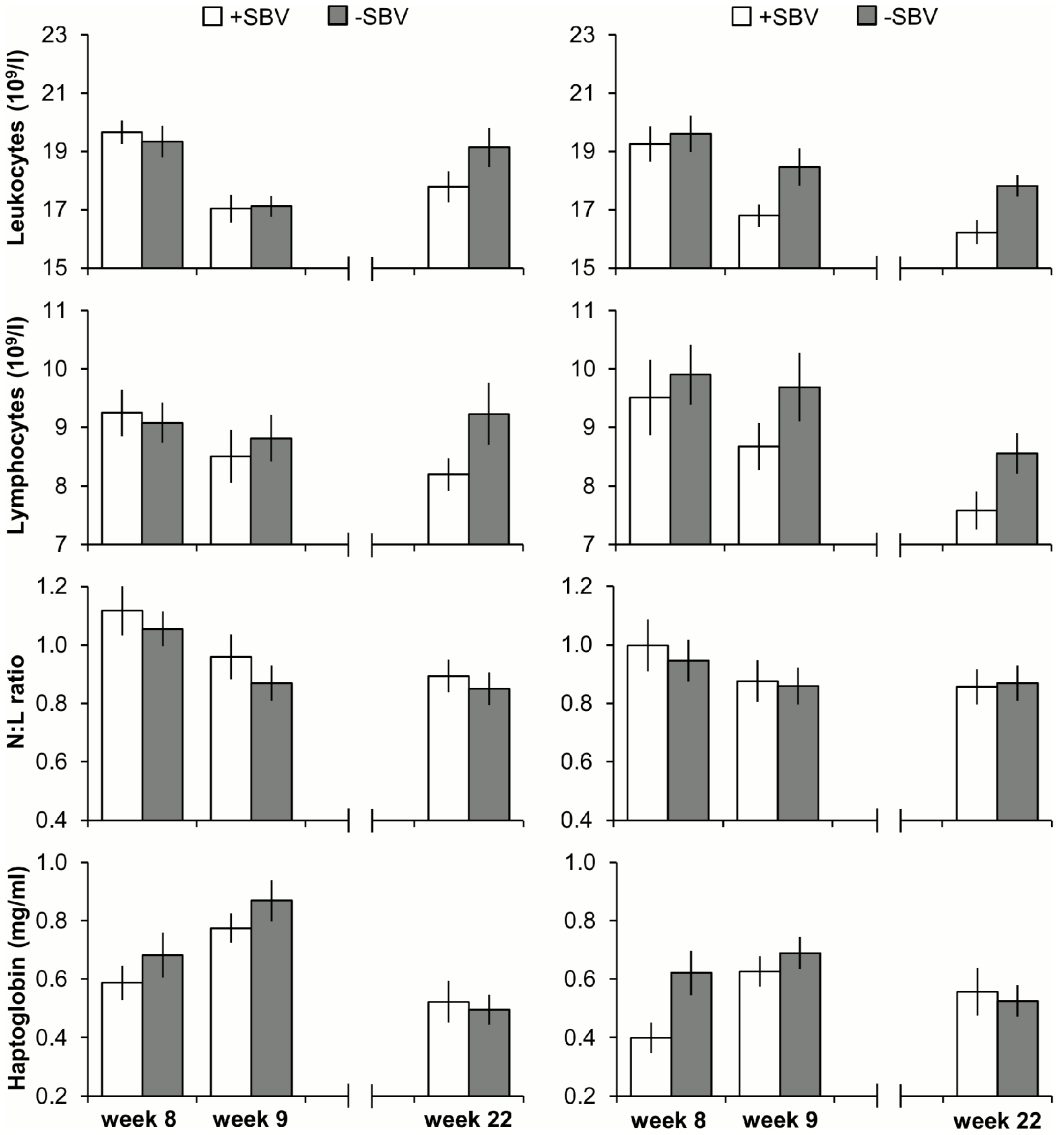
Means and SEM of leukocytes, lymphocytes, N:L ratio and haptoglobin. The concentrations of leukocytes (panels A and B), lymphocytes (panels C and D), the neutrophil to lymphocyte ratio (N:L ratio) (panels E and F), and haptoglobin concentrations (panels G and H) of pigs that have an estimated relative positive genetic effect (+SBV) or negative genetic effect (−SBV) on the growth of their pen mates in barren and enriched housing measured before a 24 h regrouping test at 8 weeks of age, after the regrouping test at 9 weeks of age and at 22 weeks of age. Significance of effects of housing and SBV is given in the text.

The concentration of leukocytes was affected by SBV class (P <0.05). Pigs with a +SBV had, overall, lower concentrations than −SBV pigs (17.8 ± 0.2 vs. 18.6 ± 0.2 10^9^/l). The concentration of leukocytes was also affected by week (P <0.001) and by the interaction between housing and week (P <0.05) (Figure 3A and 3B). Post hoc analysis revealed that leukocyte concentrations of enriched housed pigs were lower in weeks 9 and 22 compared to week 8, whereas leukocyte concentrations of barren housed pigs were lower in week 9 compared to weeks 8 and 22. In addition, leukocyte concentrations did not differ between enriched and barren housed pigs in weeks 8 and 9, but enriched housed pigs had lower leukocyte concentrations than barren housed pigs in week 22 (Figure 3A and 3B). Leukocyte concentrations were not affected by backtest classification (P  =  0.71). The decrease in the concentration of leukocytes from week 8 to 9 tended to be larger for +SBV pigs than for −SBV pigs (−2.6 ± 0.4 vs. −1.7 ± 0.4 10^9^/l, P <0.1), but was not affected by housing or backtest classification (P ≥ 0.19).

The concentration of lymphocytes was also affected by SBV class (P <0.05) and week (P <0.01) (Figure 3C and 3D). Pigs with a +SBV had lower lymphocyte concentrations than −SBV pigs (8.6 ± 0.2 vs. 9.2 ± 0.2 10^9^/l). Furthermore, lymphocyte concentrations were lower in week 22 compared to weeks 8 and 9. Lymphocyte concentrations were not affected by housing or backtest classification (P ≥ 0.22). The delta in the lymphocyte concentration between weeks 8 and 9 was also not affected by SBV class, housing or backtest classification (P ≥ 0.20).

The N:L ratio was affected by housing (P <0.05) and week (P <0.001) (Figure 3E and 3F). Enriched housed pigs had a lower N:L ratio than barren housed pigs (0.90 ± 0.03 vs. 0.96 ± 0.04). Moreover, the N:L ratio was higher in week 8 compared to weeks 9 and 22. The delta in N:L ratio between weeks 8 and 9 was not affected by housing (P  =  0.57), but was affected by the interaction between SBV class and backtest classification (P <0.05). Post hoc analysis showed, however, no differences between the groups.

### Haptoglobin

Haptoglobin concentrations tended to be affected by SBV class (P <0.1) (Figure 3G and 3H). Concentrations tended to be lower for +SBV pigs than −SBV pigs (0.57 ± 0.03 vs. 0.65 ± 0.03 mg/ml). Moreover, haptoglobin concentrations were affected by housing (P <0.01) and week (P <0.001) (Figure 3G and 3H). Enriched housed pigs had lower haptoglobin concentrations than barren housed pigs (0.57 ± 0.03 vs. 0.65 ± 0.03 mg/ml) and haptoglobin concentrations were higher in week 9 compared to weeks 8 and 22. Haptoglobin concentrations were not affected by backtest classification (P  =  0.85). The increase in haptoglobin from week 8 to 9 was not affected by SBV class, housing or backtest classification (P ≥ 0.32).

Gender did not affect any of the immune variables, except that over the three sampling points, gilts tended to have lower leukocyte concentrations than barrows (17.9 ± 0.2 vs. 18.4 ± 0.2 10^9^/l, P <0.10) and higher haptoglobin concentrations (0.62 ± 0.02 vs. 0.60 ± 0.02 mg/ml, P  =  0.1).

## Discussion

In this study, we investigated the effects of divergent selection for indirect genetic effects on growth (+SBV pigs vs. −SBV pigs) and environmental enrichment on the immune status of pigs.

In response to acute stress or inflammation, the acute phase response is activated which results, amongst others, in an increase of acute phase proteins, such as haptoglobin, and in complement activation [2], [64], [68]. The increased CPW complement activity and haptoglobin concentrations found three days after the regrouping test could, thus, indicate that the pigs experienced the test as stressful, but may also have resulted from skin inflammations caused by vigorous fighting during regrouping [50]. Moreover, pigs also had lower leukocyte concentrations and a lower N:L ratio after regrouping, whereas lymphocyte concentrations did not differ between before and after regrouping. These results are not in line with the generally reported effects of acute stress on different leukocyte types, which could be due to differences in the timing of blood sampling [59], i.e. the leukocyte levels three days after regrouping in our study may have partly reflected recovery from acute stress than the effect of regrouping stress per se.

The other effects of week on the variables measured could point to an effect of age. For instance, the increase in KLH-IgG and KLH-IgM natural antibody titers from week 8 to 9 to 22 is in line with other studies reporting rising natural antibody titers with age [62], [69]. On the other hand, APW complement activity, the concentration of lymphocytes and the N:L ratio decreased over weeks in our study. A similar result was found by Blount et al. [70] for the concentration of lymphocytes, but other studies showed a different pattern with age for these three variables [62], [71]. These inconsistencies might be due to individual variation as distinct individual variation in age-related immune changes has been reported [72].

Even though the pigs in this study were all relatively healthy and were not deliberately immunologically challenged, SBV class, housing and coping style did have clear effects on the immune variables measured. These effects were mainly found for levels of the immune variables per se and only few effects on their change due to regrouping stress. It should be stated that the amount of fresh lesions counted after regrouping did not differ between enriched and barren housing and between the two SBV classes [50], and not between the two coping styles (unpublished data). Housing affected all immune variables, except the CPW and APW complement activity and lymphocyte concentrations, and SBV class mainly affected the leukocyte, lymphocyte and haptoglobin concentrations. Moreover, effects of coping style were mainly found on the innate immune variables APW complement activity and KLH-IgG and KLG-IgM natural antibody titers. Effects of gender were also found, but these effects were rather subtle. The interpretation of these results with respect to health and (chronic) stress is, for clarity reasons, discussed in the separate sections below.

### Housing

Effects of housing were found on all variables measured except for complement activity and lymphocyte concentrations. Enriched housed pigs had, partly in line with other studies, overall a higher KLH-IgG titer [73], a lower N:L ratio [74] and lower haptoglobin concentrations [75], [76] than barren housed pigs. Enriched housed pigs also had lower leukocyte concentrations than barren housed pigs, in line with Manciocco et al. [77], but only at 22 weeks of age. On the other hand, Merlot et al. [74] found no effect of conventional or enriched housing on haptoglobin concentrations in a conventional pig breed and Manciocco et al. [77] actually found a higher N:L ratio and lower CPW complement activity in enriched housed pigs. This could be explained by differences in type of enrichment (e.g. straw, extra outdoor area, or toys), duration of enrichment provided, and age of the animals tested. The lower N:L ratio and lower haptoglobin concentrations in enriched housed pigs could indicate that enriched housed pigs were less stressed [59], [78], [79] which confirms our expectation and is in accordance with the well-established benefits of straw on behavior and welfare [17]. Straw bedding has been described as unfavorable for pathogen presence and hygiene [80] which could explain the higher circulating KLH-IgG in enriched housed pigs, but more research is needed to confirm this. All in all, the results of this study show that housing (i.e. relatively barren vs. deep straw bedding) has a substantial impact on variables related to both stress physiology and (innate) immunity which could, therefore, have consequences for both pig health and welfare. The biological significance of these effects needs to be demonstrated as pigs were not immunologically challenged. Future experimental studies addressing effects of enrichment on susceptibility to infectious challenges should reveal this, and it may be important to take the pigs' microbiome into account as well as straw enrichment has been suggested to affect gut health [81].

### Social breeding values for growth

Most notably, +SBV pigs had overall lower concentrations of leukocytes and lymphocytes and tended to have overall lower haptoglobin concentrations than −SBV pigs. In addition, the decrease in the concentration of leukocytes from week 8 to 9 tended to be larger for +SBV pigs than for −SBV pigs, suggesting that they responded differently to the 24 h regrouping test at 9 weeks of age.

Although no differences were found in the number of skin lesions after regrouping, indicating no major differences in aggression, after reunion −SBV pigs tended to act more aggressively towards their own original group members, indicating that they differently coped with the stress of regrouping. This seems to be in line with their response to other stressful situations. Previously, it was found that these +SBV pigs behaved somewhat less fearful than the −SBV pigs in several novelty tests [48], [49]. In addition, injurious biting behavior directed at pen mates (e.g. tail and ear biting) has been reported to occur less in the +SBV pens than in the −SBV pens (Camerlink et al., in press). As all pigs within a pen either had a +SBV or −SBV, these behavioral results could indicate that +SBV pigs create a less stressful social environment for themselves than the −SBV pigs. The lower leukocyte, lymphocyte and haptoglobin concentrations in the +SBV pigs support this indication and confirms our expectation, because higher leukocyte and lymphocyte levels have been associated with higher stress levels [4], [82], [83], [84] and a higher level of haptoglobin with chronic or repeated stress [78], [79]. Some caution should be made, however, because lower leukocyte and lymphocyte levels have also been associated with more stress, probably also depending on the duration of stress (e.g. acute vs. chronic stress) [59]. The observed behavioral and immunological differences between the +SBV and −SBV pigs are likely related, but in what way is, at present, not clear. It may be speculated that a higher concentration of leukocytes, lymphocytes and haptoglobin in the −SBV pigs points to a more active immune system which, in turn, could have led to an increased need of specific amino acids (e.g. for synthesis of acute phase proteins such as haptoglobin) and, thus, a reduced availability for other systems such as growth [85]. This may have stimulated these −SBV pigs to search for food and thereby have led to more biting behavior and more stress [86]. On the other hand, as all pigs in one pen were either +SBV or −SBV pigs, more biting behavior in the −SBV pens also meant receiving more bites which, likely, resulted in more inflammations and that could have led to a more active immune system in the −SBV pigs. It should be noted, though, that significantly less biting behavior was observed in the enriched pens (Camerlink et al., in press [87]), whereas the immunological differences between the +SBV and −SBV pigs were independent of housing condition (see below), suggesting that the higher leukocyte, lymphocyte and haptoglobin levels of the −SBV pigs are not the sole cause or consequence of the injurious biting behaviors. Whether the found immunological differences between the +SBV and −SBV pigs have different implications for their health is difficult to say, because both higher and lower leukocyte concentrations have been associated with better health [59] and the haptoglobin concentrations in the current study are much lower compared to haptoglobin concentrations of pigs with health problems [88]. In our study, pigs were not deliberately immunologically challenged and only few pigs became ill during the experiment, and their number did not differ between +SBV and −SBV pigs. Further research is necessary to investigate the biological significance of the differences between +SBV and −SBV pigs under less hygienic/more challenging conditions. Moreover, it is worthwhile to further investigate the health of pigs from this selection method compared with conventional selection in which selection is based on individual performance only, because one of the consequences of conventional selection has been suggested to be a heightened susceptibility to disease [89], [90].

No interactions were found between SBV class and coping style which is in agreement with earlier findings on the behavior of these pigs [48].

Interestingly, the differences between the +SBV and −SBV pigs are comparable with the differences found between enriched and barren housing: pigs housed in a better physical environment (i.e. enriched pens) may experience less chronic stress and pigs housed in a better social environment (i.e. +SBV pens) also seem to experience less chronic stress. In addition, no interactions were found between SBV class and housing condition which is in line with behavioral results of these pigs [49], [50]. This suggests that effects of this selection method on pig behavior and physiology are independent from those of housing. This is important as it may show that only a combined effort of optimizing both the breeding program and the housing environment will yield optimal results in terms of pig welfare and health.

### Coping style

In many different animal species two extremes in coping style or personality have been described: proactive and reactive [91], [92], [93]. Generally, proactive animals are aggressive, active, bold, prone to take risks and they hardly pay attention to environmental cues, whereas reactive animals are less aggressive, more cautious, avoid taking risks, but are very attentive to cues from the environment [91], [93], [94]. In pigs, similar coping styles have been distinguished based on their response in a backtest at a young age. Pigs that struggle and vocalize relatively much in this test are classified as high-resisting (HR) pigs and they resemble proactive copers, and pigs that hardly struggle and vocalize in the backtest are classified as low-resisting (LR) pigs and they resemble reactive copers [53], [54], [95], [96], [97], [98], [99], [100], [101], [102].

In this study we also found immunological differences between HR and LR pigs, even though we simply divided the population of pigs into HR or LR and did not use the extremes of the population as was done in earlier pig studies [97], [98], [103], [104], [105]. HR pigs had an overall higher APW complement activity, although the regrouping test had a more substantial effect on APW complement activity in the LR pigs. Furthermore, HR pigs also had a higher KLH-IgG titer, but only in enriched housing. At 22 weeks of age, the same result was found for the KLH-IgM titer. Together these results could indicate that on the long term HR pigs have a more active innate immune system, but that acute stress due to regrouping has a larger impact on the LR pigs. As LR or reactive pigs are more attentive to environmental cues [91], [96], a change of environment (i.e. regrouping) could indeed have more impact on them than on HR or proactive pigs. The more chronic higher innate immune activity in the HR pigs could be related to their behavior: they explore a new environment faster than LR pigs. It could be hypothesized that HR pigs are earlier exposed to pathogens than LR pigs and, perhaps, also to different pathogens. To defend themselves against these pathogens, having an active innate immune system which is the first line of defense may fit with this suggestion [106], [107]. In addition, several other pig studies investigated whether HR and LR pigs differed in specific immune responses and their results indicated that HR pigs had a stronger cell-mediated immune response, while LR pigs had a more pronounced humoral immune response [57], [58], [108]. Hessing et al. (1995) proposed that a difference in balance between T-helper 1 (Th1) and T-helper 2 (Th2) cells was underlying this difference with a shift towards Th1 in HR pigs and Th2 in LR pigs, because activation of Th1 cells leads to an inflammatory response and activation of Th2 to the production of antibodies [108], [109]. In support of this, results of an unpublished pig study by Bolhuis et al. indeed found more pro-inflammatory cytokine production in HR pigs. Furthermore, as complement activation is also associated with the activation of pro-inflammatory cytokines [110], the results of our study seem to be in line with these results. Together, these results may indicate that HR and LR pigs may have different strategies to deal with immune challenges. However, studies that investigated differences in immune responses in proactive and reactive animals are scarce and the results of these studies do not always agree with each other [111], [112], [113], [114]. In addition, in this study housing had a modulating effect on the natural antibody titers of the HR pigs, whereas Bolhuis et al. [57] found that housing had a modulating effect on specific antibody titers of the LR pigs. We propose, therefore, that more research is needed not only to be able to draw more definite conclusions about differences in immune function between proactive and reactive animals [107], but also because of the relevance of personality in actual disease susceptibility [115], [116].

### Gender

Gilts were found to have lower concentrations of leukocytes, but higher haptoglobin concentrations than barrows. The barrows were surgically castrated at 3 days of age which could explain their higher concentrations of leukocytes, because Prunier et al. [117] described that surgical castration might have long-term negative effects on the health of the pigs. The higher concentrations of haptoglobin in the gilts have also been found by others [118], [119], [120] and could indicate a fundamental difference between female and male pigs. It has been suggested that females have both a more active humoral and a more active cell mediated immune system which could be reflected in a higher susceptibility to parasites and infections in males, and females being more prone to autoimmune diseases [121], [122], [123], [124]. These differences have been attributed to differences in sex steroids, but also to differences in how females and males deal with stress on both a physiological and psychological level [121], [122], [123], [124]. As the male pigs in this study were castrated, we will refrain from speculating whether sex steroids could underlie the found immunological differences in the gilts and barrows, but differences in dealing with stress is a likely possibility, because these same gilts and barrows have been found to behave very differently in various novelty tests [48], [49] and in the regrouping test [50].

## Conclusions

Environmental enrichment is known to alleviate stress in animals. In this study, enriched housed pigs were found to have a lower N:L ratio and lower haptoglobin concentrations than the barren housed pigs which indeed suggests that enrichment has stress-reducing effects. Stress-reducing effects were also seen in pigs selected for a relatively positive genetic effect on the growth of their pen mates (i.e. +SBV pigs), because these pigs had lower leukocytes, lymphocyte and haptoglobin concentrations compared to pigs that were selected for a relatively negative genetic effect of the growth of their pen mates. Together these results indicate that both genetics and environmental enrichment can be used to improve the welfare of pigs and that the use of both together likely yields the best results.

Two effects of gender were found, but these effects were rather subtle. On the other hand, clear differences were found between pigs with a proactive or reactive coping style. Pigs with a proactive coping style seemed to have a more active innate immune status compared to pigs with a reactive coping style, pointing to a difference in dealing with immune challenges. The biological relevance of the results and their implications for health merit further research.
